# Correction: Hepatic IRE1α-XBP1 signaling promotes GDF15-mediated anorexia and body weight loss in chemotherapy

**DOI:** 10.1084/jem.2023139511052025c

**Published:** 2025-11-13

**Authors:** Yuexiao Tang, Tao Yao, Xin Tian, Xintong Xia, Xingxiao Huang, Zhewen Qin, Zhong Shen, Lin Zhao, Yaping Zhao, Bowen Diao, Yan Ping, Xiaoxiao Zheng, Yonghao Xu, Hui Chen, Tao Qian, Tao Ma, Ben Zhou, Suowen Xu, Qimin Zhou, Yong Liu, Mengle Shao, Wei Chen, Bo Shan, Ying Wu

Vol. 221, No. 7 | https://doi.org/10.1084/jem.20231395 | May 2, 2024

The authors regret that, in the original version of their article, the first paragraph of the Introduction was unintentionally duplicated from a 2020 *Cell Metabolism* article by Breen et al. cited later in the section. The authors have rewritten the first paragraph to ensure originality while maintaining the scientific context of the Introduction. The revised paragraph is shown here. This error does not affect the results and conclusions of the study. The error appears in print and in PDFs downloaded before November 7, 2025.

## Introduction

Platinum-based drugs such as cisplatin (Cis), carboplatin, and oxaliplatin are widely used in cancer therapy, but their optimal use is limited by toxicities, including nausea, vomiting, anorexia, muscle wasting, and weight loss, that compromise quality of life and treatment adherence (Kelland, 2007; Ruggiero et al., 2013). Although antiemetic regimens including 5-HT3R and NK-1 receptor antagonists, dexamethasone, and olanzapine have reduced treatment-related nausea and vomiting, breakthrough and delayed emesis remain common in many patients (Hesketh et al., 2017; Aapro et al., 2018). Mechanistic understanding of platinum-induced weight loss remains incomplete, though preclinical studies implicate NF-κB and ActRII signaling as well as ghrelin receptor activation as potential modulators (Barreto et al., 2017; Chen et al., 2017; Peterson and Guttridge, 2008). Therefore, identification of new causal mechanism(s) of chemotherapy-induced body weight loss represents a critical step to inform novel strategies for optimizing platinum treatment and improving cancer care.
